# Neural representation for distance and direction of resource for honeybees

**DOI:** 10.1186/1471-2202-12-S1-P169

**Published:** 2011-07-18

**Authors:** Sangwook Park, DaeEun Kim

**Affiliations:** 1 Biological Cybernetics, School of Electrical and Electronic Engineering, Yonsei University, Shinchon, Seoul, 120-749, South Korea

## 

Many insects manage their foraging behaviors or prey detection based on resource information. Their brain system should encode the resource location efficiently. Honeybees remember the nectar source location and communicate with their colleagues using their own dance language [1]. Desert ants demonstrate elegant homing navigation with path integration [2] and they can estimate the homing vector, distance and direction. We presume they may have common neural structure and representation for the resource vector or homing vector. For the resource vector, we can build a neural network consisting of two layers of neurons, which has a polar form representation. Each neuron is one of directionally selective neurons and also includes the information of the distance. For the distance information, the activation in direction-cell neurons has the exponential form of inhibition to the distance neuron as gate neurons, which resembles multiplicative neurons [3]. A population of the neurons efficiently encodes the resource location, that is, the distance and direction of the resource. The resource vector representation can be applied to honeybees or other insects. It is known that two types of dances in honeybees’ dance communication, waggle dances and sickle dances, has the information of resource vector. It has been pointed out that waggle dance has several cues, vibration, odor, air flow and tactile information. From the sickle dance, the colleagues on following the dancer bee can continuously records the duration and direction of its temporal path, and accumulate the information, which finally provides the net distance and direction of the resource from the comb. We suggest the mechanism is similar to the path integration system, although the accumulated distance is replaced by the accumulated duration in the preferred direction in the dance translation. The distance to the resource is involved with duration of the waggle run and duration of the return run [4]. In that point, the neural network can represent the resource information well.
